# Ultra-High-Risk Gestational Choriocarcinoma of the Ovary Associated with Ectopic Pregnancy

**DOI:** 10.3390/curroncol30020171

**Published:** 2023-02-11

**Authors:** Eva Pavla Malovrh, Nuša Lukinovič, Tatjana Bujas, Monika Sobočan, Jure Knez

**Affiliations:** 1 Faculty of Medicine, University of Maribor, 2000 Maribor, Slovenia; 2 Department of Pathology, University Medical Centre, 2000 Maribor, Slovenia; 3 Clinic for Gynaecology and Perinatology, University Medical Centre, 2000 Maribor, Slovenia

**Keywords:** gestational trophoblastic disease, ovarian choriocarcinoma, ultra-high-risk gestational trophoblastic neoplasia, ectopic pregnancy

## Abstract

Gestational choriocarcinoma of the ovary is an exceptionally rare and highly aggressive tumor. Preoperative diagnosis of extrauterine choriocarcinoma is difficult due to nonspecific clinical presentation and its resemblance to ectopic pregnancy. Without molecular genetic analysis, it is not possible to reliably differentiate gestational from non-gestational choriocarcinoma. Here, we present a case of a 44-year-old woman who presented to our emergency department with complaints of pelvic pain, vaginal bleeding, and amenorrhea. Because of a recent history of conservatively managed ectopic pregnancy, the patient underwent emergency laparoscopy. Right-sided salpingo-oophorectomy was performed due to intraoperatively suspected ovarian ectopic pregnancy. Histopathology results revealed the diagnosis of ovarian choriocarcinoma of possible gestational origin. It was classified as FIGO stage IV and WHO ultra-high-risk, and she underwent multi-agent chemotherapy without major complications. She has remained in complete remission after a 12-month follow-up. Considering the rarity of this diagnosis, we conducted a literature review including all published cases of suspected gestational choriocarcinomas of the ovary. We conclude that due to the rarity of this entity, preoperative differentiating between ovarian ectopic pregnancy and ovarian choriocarcinoma is extremely challenging, and without molecular genetic analysis, it is not possible to identify the genetic origin of the tumor.

## 1. Introduction

Gestational choriocarcinoma is a malignant neoplasm that belongs to a group of interrelated processes called gestational trophoblastic disease (GTD) [1]. GTD is characterized by abnormal proliferation of trophoblastic tissue. It includes the premalignant partial and complete hydatidiform mole and malignant invasive mole, choriocarcinoma, placental site trophoblastic tumor, and epithelioid trophoblastic tumor. Malignant variants of GTD are collectively known as gestational trophoblastic neoplasia [2,3]. 

Choriocarcinoma is the most aggressive type of GTN, and patients tend to develop early systemic metastases [4]. It can be either gestational or non-gestational in origin. However, distinguishing between gestational and non-gestational choriocarcinoma is difficult, and the tumor is generally treated without considering its origin [5]. 

Gestational choriocarcinoma usually originates in the uterine cavity, but it can rarely also affect fallopian tubes, ovaries, cervix, vagina, and abdominopelvic cavity [6,7]. It most commonly follows a molar pregnancy, but it can also develop after normal pregnancy, miscarriage, ectopic pregnancy, or termination of pregnancy [8].

Non-gestational choriocarcinoma is a rare type of tumor with trophoblastic differentiation originating from germ cells [9]. In contrast to gestational choriocarcinoma, it is unrelated to pregnancy with no paternal genes present. Non-gestational choriocarcinomas often present in children and young women. There are two types of non-gestational choriocarcinoma: the pure type, which consists of only choriocarcinoma with no other germ cell elements, and the mixed type. The latter contains other germ cell tumors such as immature teratomas or dysgerminomas [10].

Differences in gestational and non-gestational types are summarized in Table 1.

Neither gestational nor non-gestational choriocarcinoma are often considered in the initial differential diagnosis of an adnexal mass [10]. Extrauterine choriocarcinomas are exceedingly rare and can present with very aggressive clinical course; hence, timely diagnosis is crucial [6]. Due to clinical symptoms resembling ectopic pregnancy, correct diagnosis before commencing treatment is challenging [11]. These include amenorrhea, vaginal bleeding, pelvic pain, and increased serum β-hCG [12]. This means that the diagnosis of gestational choriocarcinoma is often incidental on histopathological examination after laparoscopy in suspected ectopic pregnancies [8,12].

Here, we present a case of an ultra-high-risk ovarian gestational choriocarcinoma following an ectopic pregnancy and discuss the diagnosis and treatment together with a brief review of the literature. 

## 2. Case Presentation

**Figure 1:**
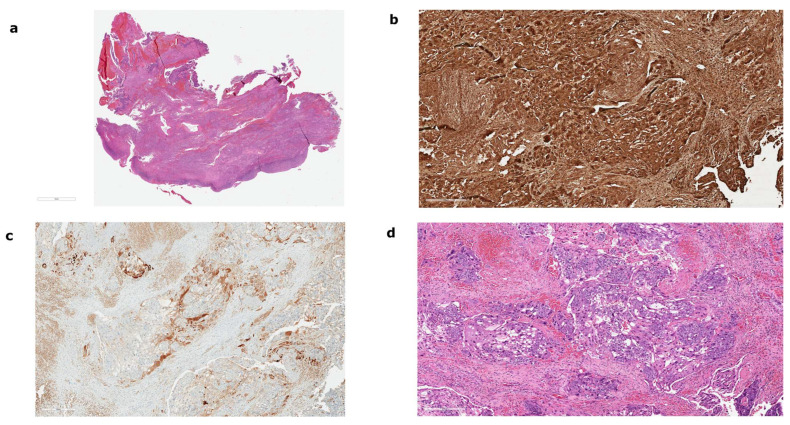
Histopathological features of the gestational choriocarcinoma. (**a**) Microscopic appearance of the ovary shows infiltration with pure gestational choriocarcinoma with widespread necrosis (H&E). (**b**) The tumor was positive for b-human chorionic gonadothropin., which is relatively specific for choriocarcinoma. (**c**) Positive inhibin stain indicates the presence of syncytiotrophoblast. (**d**) Intermediate magnification micrograph of choriocarcinoma (H&E). Choriocarcinomas consist of two cell populations: cytotrophoblast with pale/clear cytoplasm and syncytiotrophoblast with hyperchromatic cytoplasm and typically multinucleated cells.

A 44-year-old woman presented to our emergency clinic due to amenorrhea of 2 weeks, several days of vaginal bleeding, and one month history of pelvic pain. She was previously managed at our clinic 4 months before this episode due to tubal ectopic pregnancy and was then treated with an intramuscular injection of methotrexate (75 mg i.m.). In the follow-up visit one month after application, serum β-hCG level was negative. She was previously healthy without any evidence of chronic disease or any other underlying medical conditions. She had a history of one spontaneous miscarriage and one full-term pregnancy and gave birth with caesarean section five years ago. She was not taking any medications and had no known allergies. She did not use any form of hormonal contraception. Physical examination at presentation revealed abdominal pain on palpation, cervical motion tenderness and adnexal tenderness. A bloody discharge from the cervical canal was noted. Serum β-hCG was above 225,000 IU/L. The patient underwent transvaginal ultrasonography, which showed a normal uterine cavity with thin endometrium and no signs for intrauterine gestational sac or embryo. An ectopic mass of 114 × 86 × 82 mm was visualized in the right adnexal region. An area of healthy ovarian tissue was seen adjacent to the mass. Due to clinical suspicion of persistent tubal ectopic pregnancy, the patient underwent urgent laparoscopy. There was a significant hemoperitoneum and approximately 1000 mL of blood was aspirated. A large tumorous structure with pseudo-capsule was found, filling the whole pelvis. The structure was friable with a tendency to bleed on palpation. Due to excessive bleeding, the patient received a transfusion of 2 units of concentrated erythrocytes. Because the structure was indistinguishable from the ovary and because of heavy bleeding, a right-sided salpingo-oophorectomy was performed. The trophoblastic tissue was attached to the sigmoid colon, posterior uterine wall, and pelvic peritoneum. The tissue was successfully completely removed. Final histopathological diagnosis confirmed ovarian infiltration with choriocarcinoma. Histopathological case characteristics are presented in Figure 1. The pathologist could not confirm the possibility of gestational origin as no other elements of pregnancy were seen. The right fallopian tube was without signs of pregnancy or carcinoma. 

**Figure 2:**
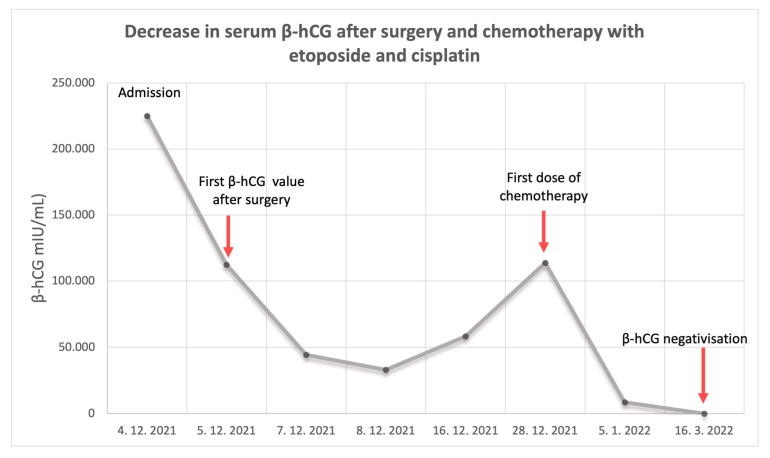
Decrease in serum β-hCG levels from admission to negativization. Biochemical follow-up was performed during the initial treatment follow as well as after systemic therapy treatment.

After surgical management, β-hCG levels abruptly declined (Figure 2). During the 12th post-operative day, β-hCG started to rise again.

Computed tomography (CT) of the abdomen, thorax and head was performed to rule out the possibility of systemic metastasis of the tumor. Chest CT revealed small parenchymal metastases in all lung segments. Because of a suspected liver lesion, magnetic resonance imaging (MRI) scan of the liver was performed, and it showed multiple suspected metastases in several liver segments, measuring up to 10 mm. The L1 vertebrum was infiltrated with 9.5 mm lesion, suspected for metastasis. Based on these findings, the multidisciplinary medical team concluded that the patient had FIGO IV stage disease. This disease was classified as ultra-high-risk according to the WHO scoring system [3]. The patient was treated with induction chemotherapy with etoposide 150 mg i.v. and cisplatin 30 mg i.v., without acute toxic side effects. After 6 cycles of subsequent etoposide, methotrexate, actinomycin D, cyclophosphamide, and vincristine (EMA-CO) regimen chemotherapy, the follow-up period was unremarkable, and there was no evidence of disease relapse after a 12-month follow-up. 

## 3. Discussion

Ovarian choriocarcinoma is an extremely rare but highly aggressive tumor that can be gestational or non-gestational in origin. Gestational ovarian choriocarcinoma can arise from an ectopic ovarian pregnancy or present as a metastasis from a uterine or tubal choriocarcinoma. Non-gestational ovarian choriocarcinoma originates from germ cells [9]. It is extremely rare, because most malignant germ cell tumors are mixed-type and consist of various malignant components, including immature teratoma, dysgerminoma, yolk sac tumor, and choriocarcinoma [15]. Our case presentation shows an ovarian choriocarcinoma that most likely developed from conservatively managed misdiagnosed ovarian ectopic pregnancy. Ovarian ectopic pregnancies represent less than 1% of all ectopic pregnancies, and conservative diagnosis based on ultrasound imaging is extremely challenging [16]. Often, the diagnosis is confirmed after final surgical management. As in our case, misdiagnosis of tubal ectopic pregnancy based on imaging is likely.

Pure non-gestational choriocarcinoma’s estimated incidence is 1:369 million and is significantly rarer compared to primary extrauterine gestational choriocarcinoma with a prevalence of 1:5335 ovarian pregnancies and 1:2.2 million normal intrauterine pregnancies. Specific prevalence of choriocarcinoma primarily originating from ovarian gestation is unknown [14].

Ovarian choriocarcinoma usually presents with irregular vaginal bleeding, abdominal pain, adnexal mass, and increased serum β-hCG. It can also present with metastatic manifestations to different organs, most commonly to the lungs and brain. Because clinical symptoms are non-specific, ovarian choriocarcinoma can be easily mistaken for other more common diseases, as in our case with ectopic pregnancy [14,17]. Gestational and non-gestational choriocarcinomas are difficult to differentiate based on history, clinical presentation, or histology [14]. In cases when the disease develops in girls prior to puberty, women who have never had sexual intercourse, or are definitely unable to conceive, we can confirm the diagnosis of non-gestational choriocarcinoma [15]. Non-gestational type choriocarcinoma involves the patients with an average age of 13 and is largely confined to females under 20 [13]. In women of reproductive age who are sexually active, gestational origin is significantly more likely. Both types of choriocarcinomas produce very high amounts of β-hCG. The value ranges from 3 to 100 times more than a normal pregnancy [7]. However, β-hCG levels are usually lower in non-gestational variants in comparison to gestational type [13]. Serum β-hCG elevation leads to pseudopuberty in premenarchal patients, while patients in reproductive age usually present with menstrual abnormalities (mainly amenorrhea) [18]. The presence of a well-developed corpus luteum of pregnancy adjacent to the tumor may also be indicative of a gestational origin. The corpus luteum or theca lutein cysts can also develop due to excess of hCG [13,19]. For definite differentiation of these two types of ovarian choriocarcinomas, DNA short tandem repeat (STR) analysis can be performed [14]. A STR is a microsatellite consisting of a unit of 2–10 nucleotides repeated several to hundreds of times, and STR analysis allows evaluation of the highly polymorphic STR loci where each individual shows differing numbers of repeated sequences of nucleotides. The genome in non-gestational choriocarcinoma comprises only a maternal (patient) allele, whereas gestational choriocarcinoma contains a paternal allele [15].

Since choriocarcinomas tend to have a good response to chemotherapy, this is the primary choice of treatment. However, the diagnosis of choriocarcinoma is commonly made after surgical removal of adnexal mass and histological examination. Surgery is also useful in controlling life-threatening hemorrhage from metastatic lesions [19]. Gestational choriocarcinoma can be treated with methotrexate, actinomycin D, or etoposide as a single agent, or with combined agents such as EMA-CO in high-risk cases. An ultra-high-risk GTN requires salvage chemotherapy in the form of low-dose induction chemotherapy consisting of etoposide-cisplatin (EP) as ultra-high-risk cases were associated with increased risk of early death either due to the tumor pathology itself or respiratory compromise and hemorrhage secondary to a heavy burden of disease or rapid tumor destruction with full-dose chemotherapy. Presently, there is very limited information about the ultra-high-risk subgroup due to its rarity [20]. In cases of resistant or relapsed disease, regimens including cisplatin such as TP (paclitaxel, cisplatin) and BEP (bleomyxin, etoposide, cisplatin) are used. When non-gestational choriocarcinoma develops as a component of mixed-type germ cell tumor, it responds well to treatment with surgery and BEP regimen. Pure-type non-gestational choriocarcinoma behaves more aggressively and is treated with cisplatin regimens [15,21]. Gestational ovarian choriocarcinoma has a better prognosis than their non-gestational counterpart. Gestational type is extremely sensitive to chemotherapy, with the overall cure rate approaching 98 % in specialized centers. However, some studies suggest that the surgical stage of pure ovarian choriocarcinoma may be a more important determinant of clinical outcome compared to being of gestational or non-gestational type [13,14].

**Figure 3:**
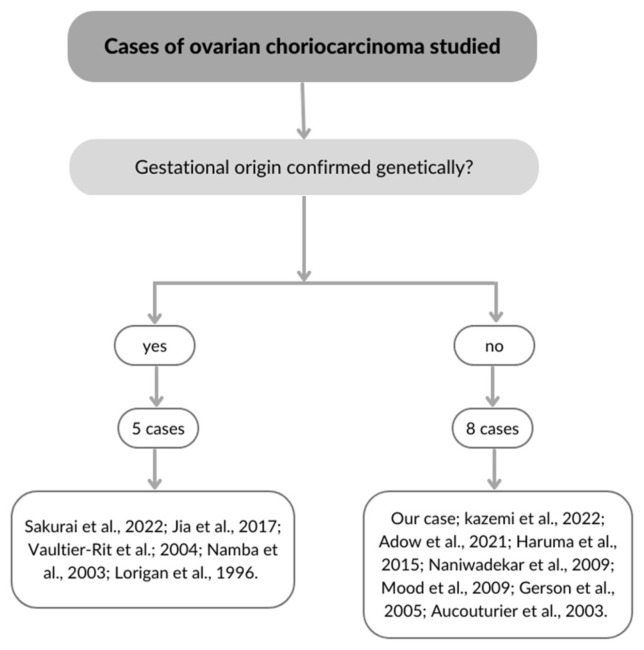
Identified cases of ovarian choriocarcinoma and their genetic origin.

We have performed a literature review and identified only 12 cases of ovarian gestational choriocarcinomas (Table 2). In five cases, the diagnosis of gestational origin was genetically confirmed (Figure 3), while in others, this was concluded based on the presence of a corpus luteum [21]. As in our case, five women were initially treated for suspected ectopic pregnancy. The remaining seven cases were already preoperatively suspected ovarian tumors. This shows that ectopic pregnancy is the main differential diagnosis, and the presentation is highly similar to extrauterine choriocarcinoma. Correct preoperative diagnosis could avoid often unnecessary surgical procedure [14]. Although staging and risk assessment based on FIGO and World Health Organization (WHO) are today the basis for management and prognosis of GTN, these were often not reported [2]. The 2000 FIGO staging system, which is the standard classification for GTN, was reported in only five cases. A modified WHO prognostic index score based on prognostic factors modified by FIGO was applied only in two cases. There was only one relapse reported out of 13 cases (7.7%), confirming excellent prognosis after standard chemotherapy treatment [21].

## 4. Conclusions

Ovarian choriocarcinoma is a challenging clinical diagnosis due to its nonspecific clinical presentation. It is commonly diagnosed after surgical management of suspected ectopic pregnancy. Gestational choriocarcinoma is significantly more common compared to non-gestational choriocarcinoma, and the origin can be suspected based on clinical characteristics, but the definitive diagnosis can only be confirmed after molecular genetic analysis. Although there are only few cases of ovarian choriocarcinomas published, the reported prognosis after chemotherapy is excellent.

## Figures and Tables

**Table 1:** Differences in gestational and non-gestational choriocarcinoma [9,13,14].

Characteristics	Gestational Type	Non-Gestational Type
Age	Reproductive period	Average age of 13 years, most patients are under 20
History of normal, molar, or ectopic pregnancy or miscarriage	Yes	No
Histology	/	Elements of other germ cell tumours are significant for mixed-type
Corpus luteum	Yes	No
Genome	Totally or partially different from the patient	Identical to the patient
Serum β-hCG	Higher	Lower
Treatment	Low-risk: single agent (methotrexate, actinomycin D or etoposide) High-risk: combination chemotherapy (e.g., EMA-CO)	Mixed-type: surgery and BEP regimen Pure type: cisplatin regimens (e.g., BEP)
Prognosis	Better	Worse (especially pure type)

EMA-CO = etoposide, methotrexate, actinomycin D, cyclophosphamide, vincristine; BEP = bleomyxin, etoposide, cisplatin.

**Table 2:** Published cases of gestational ovarian choriocarcinoma.

	Age	Clinical Presentation	βhCG (mlU/mL)	Surgery	Metastasis at the Time of Diagnosis	FIGO Grade/WHO Risk Score	Chemotherapy	Outcome	Gestational Origin Confirmed
Our case	44	Abdominal pain, vaginal bleeding	>225,000	Laparoscopic right-sided adnexectomy	Liver, lung, bone	FIGO IV WHO 16	EP, EMA-CO	complete remission	no
Sakurai et al., 2022 [5]	38	Lower left abdominal pain and abdominal distension	2.7 × 10^6^	1st surgery: left salpingo-oophorectomy and right ovarian biopsy. Artificial abortion of viable intrauterine pregnancy. 2nd surgery: total hysterectomy including the residual tumor, right salpingo-oophorectomy, and omentectomy.	no	FIGO II WHO 13	EMA-CO	complete remission	yes
Kazemi et al., 2022 [22]	35	Severe pelvic pain, fatigue, nausea, vomiting, cough	33,827	Laparotomy, not specified	lung, brain, kidney, spleen	FIGO IV	EMA-EP, EMA-CO, Relapse: 3 cycles of paclitaxel, cisplatin, etoposide, 4 cycles of liposomal doxorubicin and carboplatin, 5 cycles of fluorouracil and dactinomycin	Relapse, death 8 months from the initial diagnosis	no
Adow et al., 2021 [16]	25	Lower abdominal swelling and pain	1,000,000	Total abdominal hysterectomy and bilateral salpingo-oophorectomy	not mentioned	/	BEP	Complete remission	no
Jia et al., 2017 [21]	27	Amenorrhea, lower abdominal pain and vaginal bleeding	>200,000	Laparoscopic exploration, dissection of the cystic mass of the right ovary	no	/	EP-EMA	Complete remission, patient gave birth 25 months after chemotherapy	yes
Haruma et al., 2015 [23]	19	Lower abdominal pain, amenorrhea	373,170	Left salpingo-oophorectomy	lung, peritoneum, pelvis	FIGO III, WHO > 7 (high risk)	EMA-CO	Complete remission	no
Naniwadekar et al., 2009 [19]	19	Abdominal pain, vaginal bleeding, palpable abdominal mass	380,000	Total hysterectomy with removal of bilateral ovarian masses with omentectomy	no	/	EMA-CO	Lost to follow-up after second course of chemotherapy	no
Mood et al., 2009 [14]	31	Signs of acute abdomen and spotting	>1000	Right salpingo-oophorectomy	no	/	EMA-CE	complete remission	no
Gerson et al., 2005 [24]	33	Right lower quadrant abdominal pain	564,000	First surgery: laparoscopic right salpingo-oophorectomy and resection of a right adnexal mass Second surgery: total abdominal hysterectomy and left salpingectomy	spleen	/	EMA-CO	complete remission	no
Vautier-Rit et al., 2004 [25]	32	Pelvic pain, vaginal bleeding	315,000	Left-sided ovariectomy	no	FIGO Ic	EP	complete remission	yes
Aucouturier et al., 2003 [26]	43	Abdominal pain	37,260	Total hysterectomy with left-sided adnexectomy and omentectomy, multiple peritoneal biopsies	lung	T3c NO	EP	complete remission	no
Namba et al., 2003 [27]	37	Amenorrhea	990,000	Right salpingo-oophorectomy and a curettage of the uterus	no	/	Methotrexate, actinomycin D, cyclophosphamide as neoadjuvant therapy; methotrexate, actinomycin D, cyclophosphamide as consolidation therapy	The patient remains after follow-up with no signs of recurrence	yes
Lorigan et al., 1996 [28]	41	Amenorrhea, vaginal bleeding	151,500	Total abdominal hysterectomy, bilateral salpingo-oophorectomy, and omentectomy	no	/	BEP, salvage therapy Ifosfamide and etoposide	complete remission	yes

## Data Availability

The data presented in this study are available on request from the corresponding author.
